# Functional Diversity of Plant–Pollinator Interaction Webs Enhances the Persistence of Plant Communities

**DOI:** 10.1371/journal.pbio.0040001

**Published:** 2005-12-13

**Authors:** Colin Fontaine, Isabelle Dajoz, Jacques Meriguet, Michel Loreau

**Affiliations:** **1**UMR 7618: Biogéochimie et Ecologie des Milieux Continentaux (BIOEMCO), Ecole Normale Supérieure, Paris, France; **2**UMR 7625: Fonctionnement et Évolution des Systèmes Écologiques, Ecole Normale Supérieure, Paris, France; **3**Department of Biology, McGill University, Montréal, Québec, Canada; University of California at RiversideUnited States of America

## Abstract

Pollination is exclusively or mainly animal mediated for 70% to 90% of angiosperm species. Thus, pollinators provide an essential ecosystem service to humankind. However, the impact of human-induced biodiversity loss on the functioning of plant–pollinator interactions has not been tested experimentally. To understand how plant communities respond to diversity changes in their pollinating fauna, we manipulated the functional diversity of both plants and pollinators under natural conditions. Increasing the functional diversity of both plants and pollinators led to the recruitment of more diverse plant communities. After two years the plant communities pollinated by the most functionally diverse pollinator assemblage contained about 50% more plant species than did plant communities pollinated by less-diverse pollinator assemblages. Moreover, the positive effect of functional diversity was explained by a complementarity between functional groups of pollinators and plants. Thus, the functional diversity of pollination networks may be critical to ecosystem sustainability.

## Abstract 2

Manipulating plant and pollinator communities provides experimental evidence that the persistence of a plant community can be affected by a loss of diversity among its pollinating fauna.

## Introduction

Understanding the consequences of biodiversity loss for ecosystem functioning and services is currently a major aim of ecology [1,2]. Animal-mediated pollination is one of the essential ecosystem services provided to humankind [3,4]. The negative impact of pollinator decline on the reproductive success of flowering plants has been documented at the species level [5–7], but little information is available at the community level [8]. Increasing the scale of study to the community level is essential to account for potential competitive or facilitative effects among species that belong to the plant–pollinator network. Such effects, which are often linked to diversity [9,10], are known to have large influences on ecological processes such as community productivity and stability [11,12].

Experimental evidence for diversity effects on the functioning of terrestrial ecosystems is mainly available for plants. As primary producers, plants play a central role in the flow of energy within ecosystems [13,14]. Animal-pollinated angiosperms represent up to 70% of plant species in numerous communities and ecosystems [15]. Mutualistic interactions between animals and plants form several intricate interaction webs [16]. Recent analysis of plant–pollinator and plant–frugivore interaction webs demonstrates that these contain a continuum from fully specialist to fully generalist species [17,18]. However, these networks are structured in a nested way [19,20], with specialists mainly interacting with generalists. Such a pattern might have important consequences for ecosystem functioning, because it might confer resilience to perturbations such as the extinction of species [21] if, for example, generalist pollinators buffer the loss of specialist pollinators [18,22–24]. Furthermore, this hypothesis does not take into account the dynamical properties of these networks. In a plant–pollinator community, variations in species diversity at different trophic levels may lead to an adaptation of interaction strengths [25], which may in turn affect the total effectiveness of pollination. We conclude that more information is urgently needed concerning the impacts of biodiversity loss on multispecies and multitrophic interactions.

To experimentally test the effect of functional diversity on the functioning and persistence of plant–pollinator communities, we defined functional groups of plants and pollinators based on morphological traits. For plants, two functional groups with three species each were defined according to accessibility of floral rewards (pollen and nectar; see Figure 1). The first group (group 1) included *Matricaria officinalis, Erodium cicutarium,* and *Raphanus raphanistrum,* which have easily accessible floral rewards and will be called “open flowers.” The second group (group 2), called “tubular flowers,” included *Mimulus guttatus, Medicago sativa,* and *Lotus corniculatus,* all of which present floral rewards hidden at the bottom of a tubular corolla. For pollinators, two functional groups were defined according to mouthparts length (Figure 1). The first group (group A) included three species of syrphid flies (Diptera) with short mouthparts: *Saephoria* sp., *Episyrphus balteatus,* and *Eristalis tenax.* The second group (group B) included three species of bumble bees with longer mouthparts: *Bombus terrestris, B, pascuorum,* and *B, lapidarius*. Note that in this case a functional trait (long mouthparts) and a phylogenetic group are confounded. Preliminary observations showed that these six insect species contribute up to 70% of all pollinating visits to flowers in our study area in France. Constructing a plant–pollinator network with these four functional groups leads to a nested structure with specialists interacting with generalists (Figure 1, third column). In principle, syrphid flies cannot efficiently pollinate tubular flowers because their mouthparts are too short.

**Figure 1:**
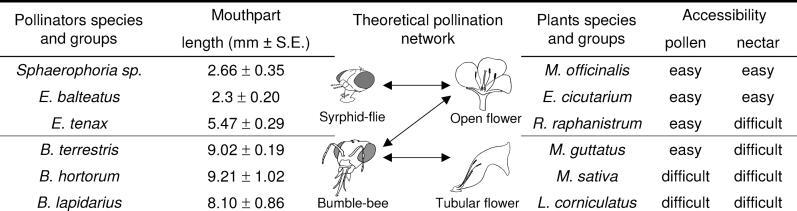
Experimental Pollination Web Summary of the characteristics upon which functional groups of pollinators (left) and plants (right) were based. In the middle, the arrows linking insect heads to flower types show the theoretical pollination network when all functional groups are present.

At the beginning of spring 2003, we set up 36 4-m^2^ caged experimental plant communities. There were three plant treatments following a “substitutive” design [26]. Two of them contained one of the two plant functional groups alone (group 1 or 2), whereas the third contained both plant functional groups in combination (group 3). We applied three different pollination treatments to each plant treatment, by introducing each pollinator functional group alone (group A or B), or both groups together (group C). This full factorial design led to nine experimental treatments, which were replicated four times each, making a total of 36 experimental units. The pollination treatments were applied in two consecutive years (June–July 2003 and 2004). We controlled for the total number of pollinator visits received by each plot during the two pollination seasons (1,000 visits in 2003 and 1,200 visits in 2004) to allow an unbiased comparison of pollination efficiency among the various experimental treatments.

In August and September 2003, we counted the number of fruits on each plant in every plot. We also counted the number of seeds per fruit on five collected fruits per plant. Lastly, in April 2004 and 2005, we measured both the number of plant species present at the seedling stage (recruitment richness) and the total number of seedlings (recruitment density) to determine the effects of the experimental treatments on the natural recruitment of the next plant generation.

## Results

### Effects on Plant Reproductive Success

The reproductive success of the two plant functional groups after the first season is analysed in Table 1. There was a significant effect of pollination treatment on the number of fruits per plant (Table 1, left; standardized means ± standard error [SE]: syrphid −0.278 ± 0.061, bumble bee 0.221 ± 0.065, and both 0.063 ± 0.068). Orthogonal contrasts on pollination treatment indicate that the identity of the pollinator guild (syrphid [A] versus bumble bee [B]) had a significant effect. There was a higher fruit production in bumble bee–pollinated communities than in those pollinated by syrphids. Moreover, the breakdown of the interaction of pollination and plant treatments into the orthogonal contrasts A1 versus B1 and A2 versus B2 indicates that the two plant functional groups responded differently to the identity of the pollinator functional group. Tubular 3 flowers (group 2) produced significantly fewer fruits in the syrphid treatment, whereas open flowers (group 1) produced the same amount of fruits whatever the identity of the pollinator functional group (Figure 2A). This supports our hypothesis that bumble bees were able to pollinate both plant functional groups whereas syrphids could only efficiently pollinate open flowers. Although the functional diversity of plant or pollinator treatment alone had no significant effect, fruit production tended to increase with both plant and pollinator functional diversity (contrast [A1 + A2 + B1 + B2] versus C3; Figure 2B).

**Figure 2:**
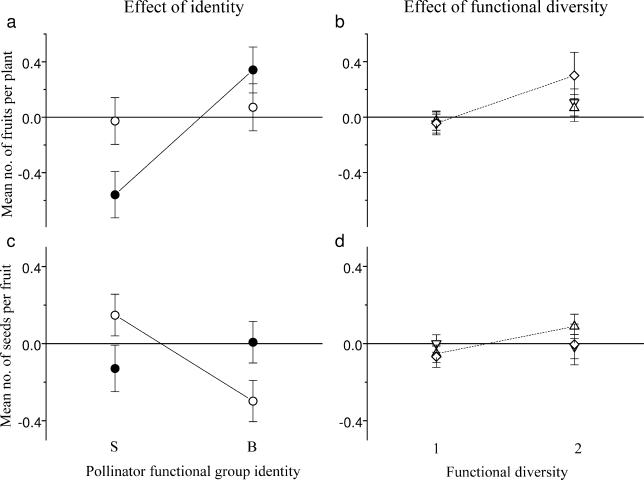
Effects of Pollinator Identity and Diversity on Plant Reproductive Success The left panels show the effects of pollinator guild identity (S indicates syrphid flies, B indicates bumble bees) on the reproductive success of the two plant guilds (open circle indicates open-flowers [group 1], closed circle indicates tubular-flowers [group 2]). Reproductive success was measured by (A) the standardized number of fruits per plant and (B) the standardized number of seeds per fruit. The right panels show the effects of the functional diversity of pollination treatments (triangle), plant treatment (inverted triangle) and both (diamond) on the standardized numbers of fruits per plant (C) and seeds per fruit (D). Lines connecting symbols indicate significant effects (solid indicates *p* < 0.001, dashed indicates *p* < 0.08). Error bars represent one standard error. See Table 1 for statistical analysis.

**Table 1:**
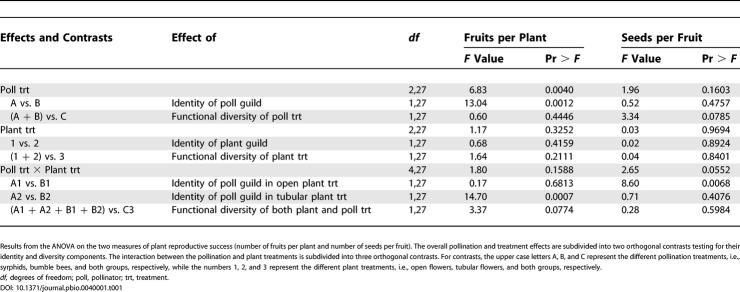
Analysis of Plant Reproductive Success

With respect to seed set per fruit, the interaction between plant and pollination treatment was marginally significant (Table 1, right). As with fruit production, the contrasts A1 versus B1 and A2 versus B2 indicate that the two plant functional groups responded differently to pollinator functional group identity. The pattern, however, was different: Open flowers produced significantly fewer seeds per fruit in the bumble bee treatment than in the syrphid treatment (Figure 2C). This means that bumblebees were less-efficient pollinators than syrphids for open flowers. This could be due to the higher rate of geitonogamous visits (i.e., consecutive visits to different flowers of the same plant, resulting in self-fertilization) by bumblebees. Indeed, preliminary observations using a similar experimental design showed that bumble bees perform a higher percentage of geitonogamous visits than do syrphids (I. Dajoz, unpublished data). Finally, the mean number of seeds per fruit in the plant communities tended to increase with functional diversity of pollination treatments (contrast [A + B] versus C; Figure 2D).

### Effects on Natural Recruitment

We analysed the long-term effects of our pollination treatments on the natural recruitment of our experimental plant treatments after the first and second pollination seasons. The results are presented in Table 2. There was a significant effect of year on recruitment richness with a higher richness after the second pollination season (mean ± SE: 1.916 ± 0.075 in 2004, and 2.291 ± 0.0856 in 2005). Among the possible causes was a severe drought in 2003 [27], which likely affected both plant and insect populations. Such a drought did not occur in 2004. This difference in climate between years may account for a large part of the year effect.

**Table 2:**
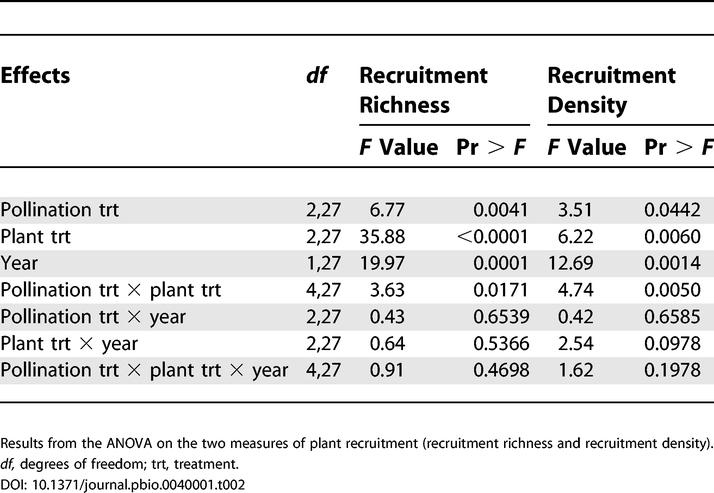
Analysis of Plant Recruitment Richness and Density

Recruitment richness was significantly different among plant treatments, with fewer species recruiting in tubular communities (Figure 3). This is very likely due to two perennial species (whereas all species are annuals in the other group) which may have different reproductive traits and create differences in competitive intensity among the plant treatments. There was a significant effect of pollination treatment, with a higher recruitment richness when both groups of pollinators were present (means ± SE: syrphid 1.854 ± 0.973, bumble bee 2.052 ± 0.826, and both 2.406 ± 1.062). However, as suggested by the significant interaction between plant and pollination treatments, the pattern was more complex (Figure 3A). In fact, pollination treatments had no effect on recruitment richness in open-flower plant treatment (Figure 3A, left). In the tubular-flower plant treatment, recruitment in the syrphid fly treatment tended to be lower than in the other pollination treatments (Figure 3A, centre). But the positive effect of pollinator functional diversity was obvious in the plant treatment that contained both plant functional groups (Figure 3A, right). In the mixed plant treatment, recruitment richness under the most functionally diverse pollination treatment was substantially above that in the two other treatments.

**Figure 3:**
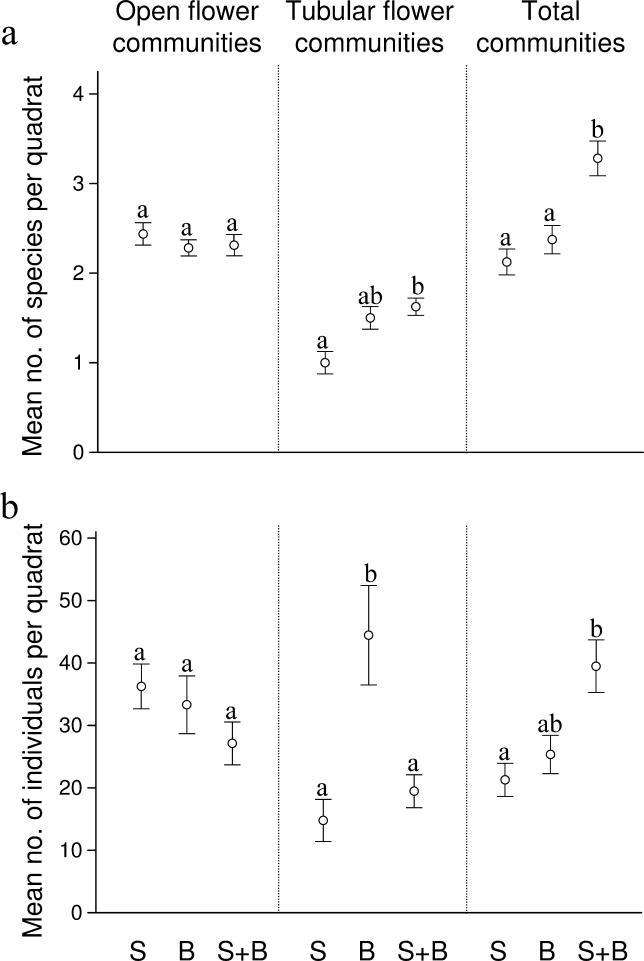
Effects of Pollination Treatments on Plant Recruitment Effects of pollination by syrphid flies (S), bumble bees (B), or both (S + B) on (A) recruitment richness (mean number of plant species present as seedlings in a quadrat) and (B) recruitment density (mean number of plant individuals present as seedlings in a quadrat) in the various plant treatments. Error bars represent one standard error. Lower-case letters indicate statistically significant differences among pollination treatments within a plant treatment (Bonferroni-adjusted *t*-test, *p* < 0.05).

Considering recruitment density, there was also a significant effect of year, with a higher density after the second pollination season (mean ± SE: 26.784 ± 2.324 in 2004 and 31.319 ± 1.937 in 2005), and a significant effect of plant treatment, with fewer individuals recruiting in tubular communities (Figure 3B, centre). These year and plant-treatment effects can be explained in the same way as for recruitment richness (see above). There was also a significant effect of pollination treatment, with a lower recruitment density when plant communities were pollinated by syrphid flies alone (means ± SE: syrphids: 24.104 ± 20.464, bumble bees: 34.364 ± 32.781, and both 28.688 ± 21.459). This is congruent with our results on the number of fruits produced per plant (see Table 1, contrast A versus B). As for recruitment richness, there was a significant interaction between plant and pollination treatments (Figure 3B). In the open-flower plant treatment, recruitment density was not significantly different among pollination treatments (Figure 3B, left). But in the tubular-flower plant treatment, recruitment density was significantly higher in the bumble bee treatment than in the other pollination treatments (Figure 3B, centre). Finally, in the mixed plant treatment, the same pattern as for recruitment richness was observed: There was a higher density in the mixed pollination treatment than in single-guild pollination treatments (Figure 3B, right).

Note that these results on natural recruitment are not an artefact caused by sampling small quadrats in heterogeneous experimental plots since the same patterns were observed when data from all quadrats in a plot were pooled.

### Pollination Visitation Web in the Mixed Plant Treatment

To explain the strong effect of pollinator functional diversity on the persistence of mixed plant communities, we carried out a log-linear analysis on the visitation rate of each insect species in a given pollination treatment, for the six plant species of the mixed plant treatment. Data from the year 2003 are illustrated in Figure 4, and the results of the analysis on both years are presented in Table 3. In the second year, there was a significant effect of plant functional group identity: Tubular flowers received a higher number of visits than did open flowers (mean visitation frequency ± SE: for open flowers 0.236 ± 0.097 and for tubular flowers 0.763 ± 0.097). This is very likely due to the two well-established perennial species, which produced a more attractive floral display during the second year of the experiment. For the two years of the experiment, there was a significant interaction between plant functional group and pollinator functional group. This indicates that the two pollinator functional groups were specialised on different plant functional groups (mean visitation frequency ± SE on open flowers and tubular flowers, respectively: in 2003, for bumble bees 0.128 ± 0.058 and 0.433 ± 0.075; for syrphids 0.327 ± 0.043 and 0.113 ± 0.052; in 2004, for bumble bees 0.01 ± 0.005 and 0.58 ± 0.075; for syrphids 0.23 ± 0.055 and 0.18 ± 0.087). Syrphids mainly visited open flowers whereas bumble bees preferentially visited tubular flowers (Figure 4). Even though bumble bees can pollinate open flowers quite efficiently when this is the only plant functional group present (as shown by the reproductive success, recruitment diversity, and recruitment density of the open-flower plant treatment in the bumble bee treatment, Figures 2 and 3), they focus on the tubular-flower group in the mixed plant treatment. In the mixed pollination treatment, the match between plant and pollinator functional groups leads to a more homogenous distribution of pollinator visits among plant groups than in the other pollination treatments. Ultimately, this significantly increases the reproductive success of plants, most likely through the homogenisation of pollinator visits and the minimization of inefficient pollinator visits.

**Figure 4:**
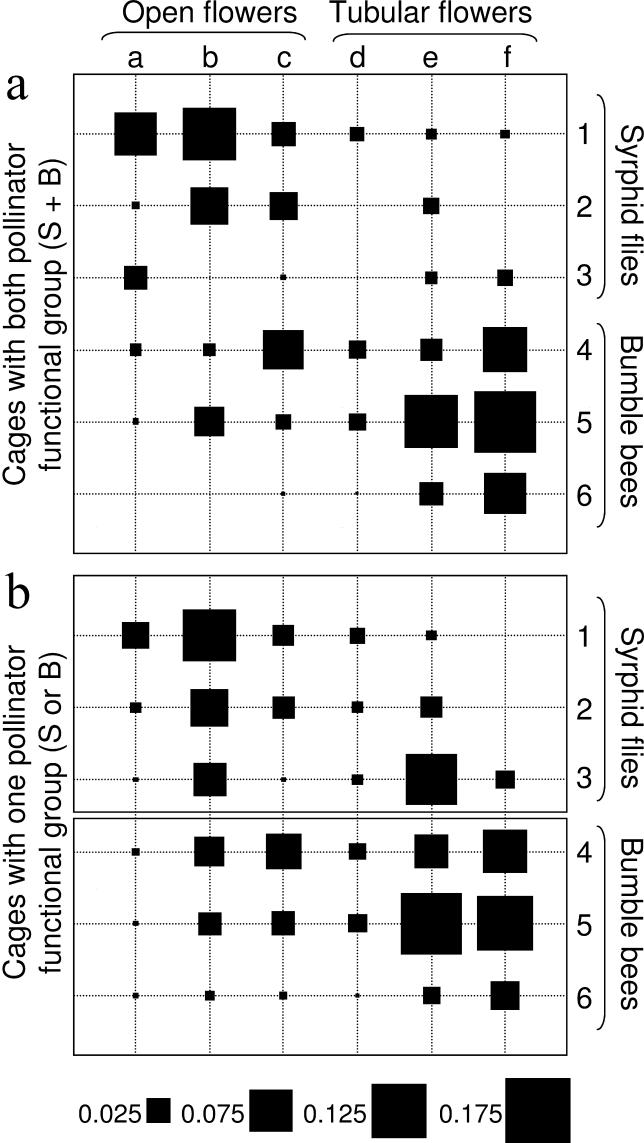
Visitation Web in the Communities with Both Plant Types Distribution of pollinator visits for the year 2003, among the six plant species in the plant treatment containing the two plant functional groups, (A) for the mixed pollination treatment (S + B) and (B) for the single functional group pollination treatments (S or B). The length of the side of the black squares shows the proportion of visits by a given pollinator species on each plant species. Lower-case letters represent plant species: a, *Ma. officinalis;* b, *E. cicutarium;* c, *R. raphanistrum;* d, *Mi.guttatus;* e, *Me. sativa;* f, *L. corniculatus*. Numbers represent pollinator species: 1, *Saephoria* sp.; 2, *Ep. balteatus;* 3, *Er. tenax;* 4, *B. terrestris;* 5, *B. pascuorum;* 6, *B. lapidarius*.

**Table 3:**
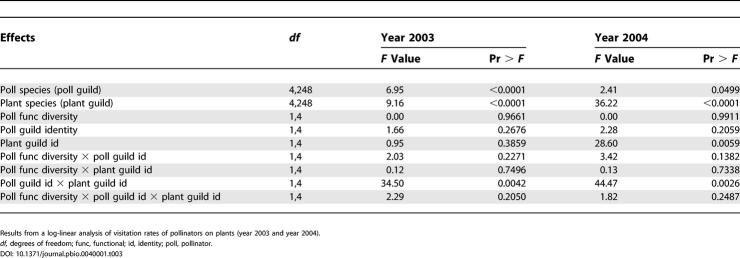
Analysis of Visitation Rates

## Discussion

Previous studies on the diversity of plant–pollinator interaction webs were either descriptive [16], carried out on a single plant species [6,7,28–30], or based on simulation [21] and theoretical approaches [22,31]. To our knowledge, this is the first experimental evidence that the persistence of a plant community can be affected by a loss of diversity of its pollinating fauna. Of course, our experimental communities differed from natural ones in several respects. Among other things, the interaction networks we studied were much simpler than those occurring in nature; in particular, they contained fewer species in each trophic level. But such simplifications from natural situations are often necessary to carry out controlled experiments.

In plant communities that contained only open flowers, plants produced fewer seeds per fruit in the bumblebee treatment than in the syrphid treatment (Figure 2C), but this was compensated by a sufficiently high fruit production, leading to a richness and density of natural recruitment that was similar to the other pollination treatments (Figure 3A and 3B left). Thus, in these communities, all pollination treatments were equally effective in the long term.

In plant communities that contained only tubular flowers, syrphids were inefficient pollinators; fruit production was very low (Figure 2A) and insufficient to allow a good natural recruitment. Bumble bees were the most effective pollinators (Figure 3A and 3C, centre). Note that in the bumble bee treatment, the very high value of average recruitment density was due to three measurements in two replicates, in which only *M. guttatus* seedlings were recorded at a very high density (more than 150 seedlings per quadrat). To test the effect of these outliers, we removed them and repeated our analysis. The same significant effects were observed, except for the effect of pollination treatment, which became marginally significant (*p* = 0.0645). The new mean number of seedlings per quadrat for this experimental treatment was 32.17 ± 4.55 (SE), which is still slightly above the value for the pollination treatment with both pollinator groups. For plant communities that contained only tubular flowers, recruitment richness in the two pollination treatments that contained bumblebees was similar.

These results are in agreement with our theoretical pollination network presented in Figure 1. In our experimental system, syrphids can be considered as specialist pollinators since they efficiently pollinate only open flowers. Bumble bees were potentially generalists as they induced an important fruit production of the two plant types and a good recruitment in the open- and tubular-flower plant treatments. Our results on the reproductive success and recruitment of single-guild plant treatments indicate that there are strong functional group identity effects since our plant functional groups responded differently to our pollinator functional groups.

However, the functional diversity of both the plant and pollination treatments was also important. Plant reproductive success tended to increase with pollinator functional diversity when the number of seeds per fruit was considered, and with both plant and pollinator functional diversity when the number of fruits per plant was considered (Figures 2B and 2D). Although recruitment in single-guild plant treatments was mainly affected by the identity of functional groups, the effect of functional diversity was dramatic in the mixed plant treatment. Natural recruitment of plant communities visited by mixed pollinator guilds was largely above that in other pollination treatments.

Pollination by syrphids alone allowed the reproduction of open flowers but not tubular flowers, as expected from the specialisation of syrphids. More surprisingly, however, bumble bees failed to be efficient generalist pollinators. Most of their visits occurred on tubular flowers (Figure 4), resulting in a relatively poor recruitment of open flowers. The only pollination treatment that achieved a high recruitment of both open and tubular flowers when they were mixed, was the one containing the two insect functional groups (Figure 3, right). When syrphids and bumble bees simultaneously pollinated mixed plant communities, they each focused on their target plant functional group, leading to more efficient visits and a better distribution of visits among plant functional groups (Figure 4). Ultimately, it was the pollination treatment with both pollinator functional groups that produced the highest richness and density of natural recruitment. Consequently, since most natural plant communities contain both open and tubular flowers, pollinator functional diversity should strongly enhance the persistence of these communities.

Although our experimental system differed from natural communities, and information about the reciprocal effects of the functional diversity of plant communities on the diversity of pollinator communities would be useful, our study indicates that the functional diversity of plant–pollinator interaction webs may be critical for the persistence and functioning of ecosystems and should be carefully monitored and protected. The loss of pollinator functional diversity is likely to trigger plant population decline or extinctions [4], which in turn are likely to affect the structure and composition of natural plant communities and the productivity of many agroecosytems that rely on insect pollination [8]. Ultimately, higher trophic levels may be affected since the diversity and biomass of consumers depend on primary production. Our results strongly suggest that the functional diversity of complex interaction webs plays a crucial role in the sustainability of ecosystems.

## Materials and Methods

### Experimental plant communities

At the beginning of spring 2003, plant communities were set up in a meadow that remained almost undisturbed for 10 years at the Station Biologique de Foljuif, France, 80 km southwest of Paris. Prior to the establishment of the communities, soil was sterilized by injecting 120 °C steam (30 min) to destroy the seed bank and soil pathogens. In each of the 36 4-m^2^ plots, a total of 30 adult plants were planted on a grid, spaced 25 cm from each other, to minimize competition and homogenise spatial distribution. Thus, plant density was the same in all experimental plots. We selected a moderate density to maintain within- and among-species competition to a low level, and to allow enough space for future recruitment in the plots. Each of these plant communities was enclosed in a 2-m–high nylon mesh cage in order to eliminate natural pollinator visitation.

### Pollination rounds

During the flowering seasons (June–July 2003 and 2004), pollinators were captured around the study area and introduced into the cages. The relative abundance of pollinator species in the various pollination treatments reflects their natural abundances. From preliminary observations, we had noticed that, in order to have no more than three insects active at the same moment in a 4-m^2^ plot, it was necessary to put about eight syrphid flies, or six bumble bees, or a mixture of six syrphids and four bumble bees in each pollination cage. Each pollination round in a given plot included 200 visits in the year 2003 and 300 in the year 2004. In total, each plot received either four (in 2004) or five (in 2003) pollination rounds, leading to a total of 1,000 visits per plot in 2003 and 1,200 in 2004.

### Pollination activity

Bumble bees needed approximately 30 min after introduction in the cages to calm down and start to pollinate. In the pollination treatment with both pollinator guilds, we then introduced syrphids, which started to pollinate immediately. Mean visitation time was not significantly different between insects in the cages and in nature. This was true both for bumble bees (mean visitation time in cages: 3.25 ± 0.92 s, mean visitation time in nature: 2.91 ± 1.33 s, *t =* 1.51, *df* = 96, *p* = 0.133) and for syrphids (mean visitation time in cages: 40.21 ± 8.89 s, mean visitation time in nature: 35.38 ± 14.75 s, *t =* 0.77, *df* = 12, *p* = 0.45).

### Measurement of reproductive success

One month after the first pollination treatments, we counted the total number of fruits on each plant, except for *M. guttatus* and *M. officinalis* in which fruits cannot be counted without collecting them. We randomly took five fruits per plant of each species to estimate the number of seeds per fruit.

### Measurement of recruitment richness and density

Recruitment richness and density were estimated during the second (April 2004) and third (April 2005) year of the experiment by counting the number of seedlings of each species in four 1,600-cm^2^ quadrats in each plot.

### Statistical analysis

Statistical analyses were performed using SAS 8.2 software.

For the analysis of plant reproductive success, we log-transformed the data to ensure normality. We standardized the data by species using the formula: *x* − μ/σ (where μ = the mean and σ = the standard deviation of number of fruits or number of seeds per fruit for a given plant species) in order to make the data comparable among the various species and functional groups. We used a mixed analysis of variance (ANOVA) model (SAS proc mixed), in which the fixed effects were plant treatment, pollination treatment, and their interaction term. To investigate the effects of the various plant and pollination treatments, we subdivided a priori each main effect into two components using orthogonal contrasts. The first contrast tested the effect of the identity of the plant or pollinator functional group, i.e. one group versus the other. The second tested the effect of the functional diversity of the plant or pollination treatment, i.e. single-guild versus mixed-guild plant or pollination treatments. Similarly, we subdivided the interaction into three orthogonal contrasts testing the effects of pollinator functional group identity on each plant guild, and the effect of the functional diversity of both plant and pollination treatments. See Table 1 for the construction of the contrasts.

For the analysis of plant recruitment, we used a repeated measure ANOVA model (SAS proc mixed). The fixed effects were pollination treatment, plant treatment, year, and all the interaction terms. The repeated effect was year, and the subject effect was replicate. For recruitment density, data were log transformed.

For each year of the experiment, the visitation rate of pollinators on each plant species in the communities with both plant functional groups was analysed using a mixed log-linear model (glimix macro, SAS). We subdivided the pollination treatment into two effects: pollinator functional diversity (one or two pollinator functional groups) and identity of the pollinator functional group (bumble bees or syrphids). The model included pollinator species nested within identity of pollinator functional groups, plant species nested within identity of plant functional group, identity of pollinator functional groups, identity of plant functional groups, pollinator functional diversity, and all interaction terms. The replicate was a random effect.

## Abbreviations

A: syrphid group
ANOVA: analysis of variance
B: bumble bee group
C: combined (syrphid and bumble bee) group
df: degrees of freedom
group 1: open flower
group 2: tubular flower
group 3: combined (open and tubular) flower
SE: standard error
